# Utilization of wearable technology to assess gait and mobility post-stroke: a systematic review

**DOI:** 10.1186/s12984-021-00863-x

**Published:** 2021-04-21

**Authors:** Denise M. Peters, Emma S. O’Brien, Kira E. Kamrud, Shawn M. Roberts, Talia A. Rooney, Kristen P. Thibodeau, Swapna Balakrishnan, Nancy Gell, Sambit Mohapatra

**Affiliations:** Department of Rehabilitation and Movement Science, University of Vermont, 106 Carrigan Dr., Rowell 310, Burlington, VT USA

**Keywords:** Stroke, Wearable, Gait, Mobility, Rehabilitation, Sensors

## Abstract

**Background:**

Extremity weakness, fatigue, and postural instability often contribute to mobility deficits in persons after stroke. Wearable technologies are increasingly being utilized to track many health-related parameters across different patient populations. The purpose of this systematic review was to identify how wearable technologies have been used over the past decade to assess gait and mobility in persons with stroke.

**Methods:**

We performed a systematic search of Ovid MEDLINE, CINAHL, and Cochrane databases using select keywords. We identified a total of 354 articles, and 13 met inclusion/exclusion criteria. Included studies were quality assessed and data extracted included participant demographics, type of wearable technology utilized, gait parameters assessed, and reliability and validity metrics.

**Results:**

The majority of studies were performed in either hospital-based or inpatient settings. Accelerometers, activity monitors, and pressure sensors were the most commonly used wearable technologies to assess gait and mobility post-stroke. Among these devices, spatiotemporal parameters of gait that were most widely assessed were gait speed and cadence, and the most common mobility measures included step count and duration of activity. Only 4 studies reported on wearable technology validity and reliability metrics, with mixed results.

**Conclusion:**

The use of various wearable technologies has enabled researchers and clinicians to monitor patients’ activity in a multitude of settings post-stroke. Using data from wearables may provide clinicians with insights into their patients’ lived-experiences and enrich their evaluations and plans of care. However, more studies are needed to examine the impact of stroke on community mobility and to improve the accuracy of these devices for gait and mobility assessments amongst persons with altered gait post-stroke.

**Supplementary Information:**

The online version contains supplementary material available at 10.1186/s12984-021-00863-x.

## Introduction

Use of wearable technologies has become more prominent in both community and healthcare settings with advancements in technology and the increased need for telehealth [1]. As the link between physical inactivity, morbidity, and mortality has become increasingly understood [2], researchers have begun to utilize wearable technology to examine walking and physical activity metrics amongst populations of interest, including persons with stroke [3–14]. Wearable activity monitors and pedometers have been widely used to examine physical activity levels, predominantly via assessment of daily step count and step rate. Commonly used consumer-grade wearable technologies include Fitbit, Apple Watch, and Garmin VivoSmart HR + . Research-grade wearable technologies include the Actigraph GT3X + and ActivPAL [15]. Although consumer-grade devices have been shown to overestimate and underestimate physical activity levels compared to research-grade devices, the overall correlation between activity trackers is high [16, 17]. Moreover, consumer-grade devices are less expensive and more user-friendly than what is currently being used in lab settings [18]. Additionally, the accessibility of such wearable sensors may benefit the translation of lab-centered research to the household and community level, as snapshots of mobility in a controlled research setting do not always reflect a person’s mobility in their day-to-day life. Home or community-based assessments of walking speed and distance via wearable technology can provide ongoing insight into a person’s functional performance post-stroke.

Consequences of stroke including lower extremity weakness, post-stroke fatigue, postural instability, and cognitive impairment often contribute to gait and mobility deficits [19, 20]. During the acute phase post-stroke, increased time in bed has been found compared to the subacute phase, with sitting times similar between phases [15]. Additionally, more than half of chronic stroke survivors continue to experience walking deficits and reduced mobility [21, 22]. Wearable technologies afford an exciting avenue to monitor and provide feedback on walking function across the different phases of stroke recovery. Such insight can be used by clinicians to inform and/or modify plans of care and treatment approaches, and can be used by patients to effectively self-monitor progress during and after stroke rehabilitation. For example, real time feedback and data visualization, accessible by patients and rehabilitation providers, can indicate a sustained decrease in daily physical activity thereby prompting a reassessment and treatment plan for contributing factors. Similarly, wearable data can provide a crosswalk of sorts between improvements in rehabilitation metrics, such as strength and balance, and changes in community mobility and physical activity. Early identification of a mismatch between gains achieved in rehabilitation and community mobility is a novel metric that can be used to modify rehabilitation interventions accordingly.

One of the most easily measurable, reliable, and sensitive ways to assess mobility deficits in persons post-stroke is gait speed [23]. Gait speed has been described as the sixth vital sign and is a predictor of independence, mortality, functional status at home and in the community, and quality of life (QOL) [24]. Gait speed can also be used to stratify patients into functional ambulation classifications [household ambulator (< 0.4 m/s), limited community (0.4–0.8 m/s), and full community ambulator (> 0.8 m/s)], with improvements in speed-based gait classifications associated with improved function and QOL in persons with stroke [25]. There is great variability in how gait speed is measured in research and rehabilitation, with different walking distances (e.g., 3-, 4-, 10-m), protocols (static versus dynamic starts/stops), speed (self-selected versus fast), and instructions used [26, 27]. The 10-m walk test is commonly considered the gold standard for gait speed assessments [28]. Post-stroke impairments in hip power generation and ankle plantarflexor force production can significantly affect gait speed [29]. Other common spatiotemporal deficits include decreased paretic stance time and decreased step length, resulting in asymmetrical gait patterns and decreased cadence [30–32]. Due to the relationship between these variables and gait speed, assessment of kinetic forces and key spatiotemporal parameters of gait via wearable technology could help target rehabilitation intervention strategies to improve walking post-stroke.

Emerging wearable technologies can provide new opportunities to enhance assessment and rehabilitation post-stroke. The number of stroke survivors is growing due to earlier detection and improved medical interventions, yet many continue to live with disability [33]. It is impossible for healthcare systems to adequately monitor these chronic stroke survivors long-term and identify early signs of physical and/or functional decline. Wearable devices allow the capturing of mobility and physical activity performance in different free-living settings, and clinical access to this data can potentially assist with earlier identification of functional decline and improve timeliness of referrals, reassessment, and treatment [34, 35].

To our knowledge, there is limited research on the use of wearable technology to assess gait and mobility post-stroke. A majority of the available research includes intervention studies conducted in laboratory and inpatient rehabilitation settings that have used sensors to investigate change in cadence, step time variability, and gait speed [6, 12, 13, 36]. Other studies have used sensors as an intervention tool. For example, results from Mansfield et al. showed that providing physical therapists with activity data from a wearable device led to increased focus on ambulation intensity and gait speed during post-stroke inpatient rehabilitation [36]. While these studies were conducted in more idealized laboratory or clinical settings, the utility of such data is often not sufficient in assessing or predicting an individual's true functional mobility and recovery post-stroke. A return to home and community-based ambulation is commonly one of the primary goals during stroke rehabilitation, as it relates to overall activity, participation, and health [37]. As wearable technologies continue to progress in affordability and accessibility, such technologies can enable the gathering of movement-related data in "real-world" settings, providing insight into the lived experiences of individuals with stroke that can inform rehabilitation providers and guide intervention strategies. Most consumer-grade wearable devices (such as Fitbit) are more affordable than research-grade wearables (such as ActivPAL), with research-grade wearables increasingly being used alongside or instead of expensive lab equipment such as force plates and 3D motion analysis systems for the assessment of gait [38, 39].

Wearable technologies can provide researchers and clinicians valuable information to guide interventions, as well as help to inform best practices and prevention efforts. Technologies such as wireless sensors, accelerometers, gyroscopes, pressure sensors, and personal activity monitors (combined with machine-learning algorithms) have allowed for the measurement and monitoring of gait and mobility amongst the general public and in specific patient populations [3–5, 7–14]. While a variety of wearable technologies are available, not all enable accurate and reliable measurement in patients who present with atypical gait [6]. The psychometric properties of accelerometers, pedometers and inertial measurement units primarily have been validated in healthy populations. The accuracy and reliability of these devices in capturing gait and mobility metrics of pathological gait is unclear. A limited number of studies have examined the efficacy of specific sensors and their ability to accurately report spatiotemporal parameters of gait and gait events in persons post-stroke [40]. Gait abnormalities such as inconsistent or slow stepping/walking speed and decreases in single limb stance time can contribute to fluctuating walking accelerations that can limit the accuracy of some sensors (e.g., Opal single IMU worn at the lumbar spine, Fitbit Zip worn at the non-paretic hip, ActivPAL worn on the paretic leg) to capture variation in gait events in persons post-stroke [40–42]. Thus, information on the validity and reliability of specific wearable devices is needed to gauge their ability to accurately capture various walking metrics in persons with stroke who exhibit more impaired gait deficits.

Impaired gait and mobility post-stroke often have far-reaching effects and can dramatically impact social reintegration, life satisfaction, and community mobility [43–46]. Integration of persons post-stroke into the local community is warranted to help promote functional independence and QOL. Recent studies have demonstrated the significance of assessing not only gait impairments post-stroke, but also life-space, community mobility, and QOL as such information can provide a richer understanding of the impact of impaired mobility on the lives of persons with stroke [45]. Advances in wearable technology, in combination with outcomes collected from global positioning system devices, ecological momentary assessment, and SenseCams, provide a unique means for in-depth assessments of gait and mobility post-stroke. Furthermore, examining relationships between wearable technology-derived gait and mobility variables and patient-reported health outcomes (e.g., Stroke Impact Scale, Activities-Specific Balance Confidence scale) may help identify barriers contributing to reduced mobility post-stroke and clarify the impact of gait interventions on overall recovery.

As wearable technologies continue to advance and become more accessible, their potential for use in rehabilitation research and clinical practice will grow. In order to improve the utility of wearable technology for assessing and improving mobility post-stroke, a better understanding of how this technology has been used to assess gait and mobility post-stroke is needed. Thus, the purpose of this systematic review is to evaluate how and in what settings wearable technologies, such as consumer and research-grade wearable devices, have been used for assessment of gait and mobility in individuals post-stroke.

## Methods

### Defining wearable technology

Building on the work of Godfrey et al. [64] and Parker et al. [65] in defining wearable technology in the context of post-stroke rehabilitation, we used the following for the current study: “Wearable technology encompasses any wearable device that is worn externally on the body, is wireless, and captures parameters related to movement and gait. Wearable technology is not limited to the laboratory environment and may be used in free-living conditions.” We note that mobile phones, although not exclusively “wearable technology,” may be used in this capacity by extracting accelerometer data collected by the phone while worn on the body. Accordingly, we included “mobile phones” in the search strategy for studies that used them as wearable technology.

### Search strategy

For this systematic literature review we followed the recommended steps as described in Khan et al. [47]. The focus of this review was on journal articles published in English from 2010 up to September 30, 2020 that described the use of wearable technology to assess gait and mobility in persons post-stroke. PRISMA guidelines were used [48]. We searched the following databases: Ovid MEDLINE® (Medical Literature Analysis and Retrieval System Online), CINAHL (Cumulated Index to Nursing and Allied Health Literature), and Cochrane Trials. Our PICO criteria included the following: P (Population): stroke, I (Intervention): wearable technology, C (Comparator): not applicable, O (Outcome): gait and mobility. To find articles related to our PICO we used the following MeSH (Medical Subject Heading) terms: “stroke” OR “stroke rehabilitation” OR “cerebrovascular disorders” AND “wearable electronic devices” OR “fitness trackers” OR “cell phone” OR “monitoring/ambulatory” OR “accelerometry” AND “gait” OR “activities of daily living” OR “exercise” NOT “robotics” OR “exoskeleton”. Our search also included the following keywords: “stroke” OR “cerebrovascular accident” OR “cva” (cerebrovascular accident) AND “wear activity tracker” OR “wear electronic device” OR “wear diagnostic device” OR “wear computer device” OR “fitness tracker” OR “activity tracker” OR “cell phone” OR “cell telephone” OR “mobile phone” OR “mobile telephone” OR “accelerometer” OR “ambulatory monitor” OR “outpatient monitor” OR “microcomputer” OR “smartphone” OR”inertial measure unit” OR “imu” (inertial measurement unit) OR “gyroscope” OR “smart watch” OR “pedometer” OR “gps” (global positioning system) AND “activities of daily living” OR “adl” (activities of daily living) OR “exercise” OR “physical activity” OR “walk”, OR “resistance” OR “aerobic” OR “endurance” OR “ambulation” OR “gait”. A detailed search strategy on how the searches were conducted with exact search strings is attached as an Additional file 1: Appendix.

### Eligibility criteria

Studies were included if they were conducted on persons with stroke (≥ 18 years of age) within any time frame post-stroke (i.e., acute, subacute and chronic) and investigated the use of wearable technology in relation to the assessment of walking and mobility post-stroke. Exclusion criteria included the following: (1) studies that were not written in English, (2) studies that were published prior to 2010, (3) systematic literature reviews, (4) protocol studies that did not contain any data or results, (5) studies that used wearable technology only as a modality for treatment, (6) studies that solely looked at upper extremity function and mobility, and (7) studies conducted on children (≤ 18 years of age). Additionally, articles using “exoskeletons” or “robotics” were excluded since these forms of technology are primarily used to promote movement rather than assess it.

### Study selection

All studies identified from the databases were compiled and uploaded to Zotero reference management software (https://www.zotero.org; Corporation for Digital Scholarship, USA) for review, at which point we removed duplicates. Initial title and abstract screening eliminated studies based on inclusion and exclusion criteria, and was carried out by two authors per study (K.K., E.O., S.R., T.R., or K.T.). Studies that were not eliminated were reviewed further in full text against inclusion and exclusion criteria by the same two authors, independently. The first author (D.P.) resolved discrepancies in determining if an article met eligibility criteria.

### Quality assessment

A minimum of two reviewers (K.K., E.O., S.R., T.R., or K.T.) completed the quality assessment of each included article using the Physiotherapy Evidence Database (PEDro) quality scale for clinical trials [49] and the Strengthening the Reporting of Observational studies in Epidemiology (STROBE) checklist for cross-sectional studies [50]. The PEDro scale is a checklist of 11 items that are used to evaluate the quality of clinical trials by using yes or no statements that are scored based on whether they are stated in the article or not. This scale looks at the external and internal validity of the randomized clinical trial being evaluated as well as statistical information. The STROBE checklist consists of 22 items evaluating the methods, results, and other distinguishing features of a cross-sectional study. The checklist does not determine quality grades; however, a higher score is associated with a better quality study.

### Data extraction

We extracted data that included study author name(s) and year, number of subjects, subject demographics such as age and gender, time post-stroke, side of stroke (left or right), assistive device use, environment for data collection (lab-based, community, etc.), type(s) of wearable device used, location the device was worn on the participants body, gait variable(s) or parameters examined, and main findings for primary and secondary outcomes. We also extracted, when applicable, statistical analyses (e.g., p-values, correlational values) and wearable technology reliability and validity metrics.

## Results

### Search results

**Fig. 1:**
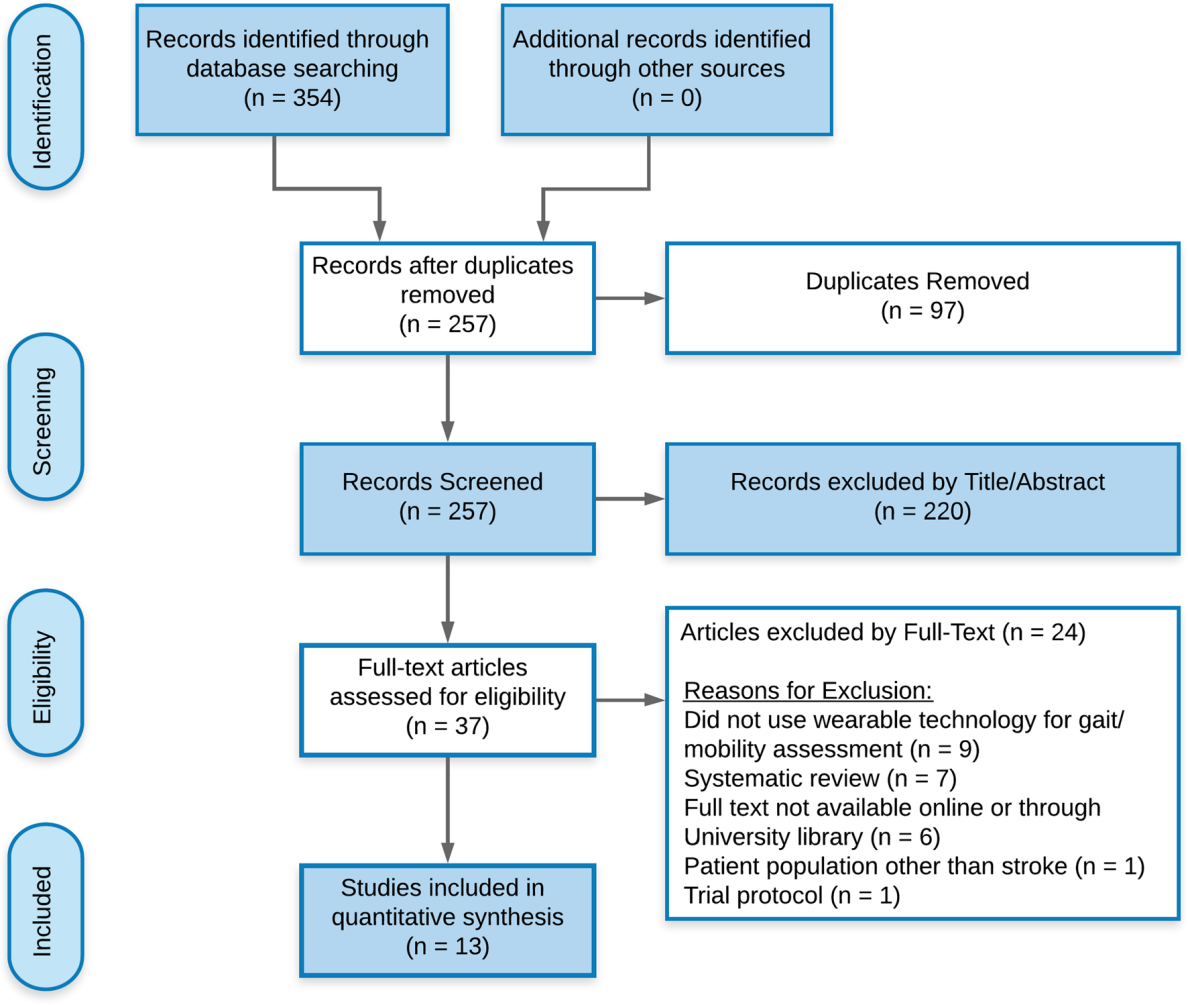
PRISMA flowchart for systematic review

We identified a total of 354 articles via our initial database search. 97 duplicates were removed after initial screening. The titles and abstracts of 257 remaining articles were further screened. Of those, 220 articles were excluded as they contained at least one exclusion criteria. If the reviewers were unable to find at least one exclusion criteria during the title and abstract screening, a full-text review of the article was warranted. The number of full texts articles assessed for eligibility was 37. Following full-text review, we excluded another 24 articles. The remaining 13 articles [36, 42, 51–61] met inclusion criteria and were included in this systematic review (see Fig. 1 for PRISMA flowchart). Tables 1, 2, 3, 4, 5 outline study characteristics, quality assessment, wearable technologies utilized, and data reported.

**Table 1:** Type and quality of included studies

Article	Type of study	Environment of data collection (lab-based, inpatient, outpatient, community, or combination)	Level of evidence	Quality of evidence
Dorsch et al. [51]	Randomized Control Trial	Inpatient	2	High
Mansfield et al. [36]	Randomized Control Trial	Inpatient	2	High
English et al. [52]	Randomized Control Trial	Community	2	High
Givon et al. [53]	Randomized Control Trial	Not explicitly stated. Interventions provided by occupational therapists in a clinical setting	2	High
Danks et al. [54]	Randomized Control Trial	Outpatient clinical research laboratory	2	High
Kanai et al. [55]	Randomized Control Trial	Hospital	2	High
Prajapati et al. [56]	Cross-Sectional	Hospital	4	Moderate
Taraldsen et al. [42]	Cross-Sectional	Hospital	4	Moderate
Tramontano et al. [57]	Cross-Sectional	Hospital	4	Moderate
Wang et al. [58]	Cross-Sectional	Hospital	4	Moderate
Seo et al. [59]	Cross-Sectional	Not explicitly stated. Subjects were persons with chronic stroke	4	Moderate
Paul et al. [60]	Pilot Study: Non-randomized control trial	Community	3	Moderate
Shin et al. [61]	Longitudinal pilot study	Inpatient/outpatient	4	N/A

**Table 2:** PEDro scale for clinical trials

Author	Eligibility criteria specified^a^	Subjects randomly allocated to groups	Allocation was concealed	Groups were similar at baseline	Blinding of all subjects	Blinding of all therapists who provided therapy	Blinding of all assessors	Measures at least 1 key outcome obtained from > 85% of subjects	All subjects received allocated tx or control or “intent to treat” analysis	Results of between group statistical comparisons are reported	Study provides both point measures and measures of variability	Total
Dorsch et al. [51]	1	1	1	1	0	0	1	0	0	1	1	6
Mansfield et al. [36]	1	1	1	1	0	0	1	1	0	1	1	7
English et al. [52]	1	1	1	1	1	0	1	1	1	1	1	9
Givon et al. [53]	1	1	1	1	0	0	1	1	1	1	1	8
Danks et al. [54]	1	1	1	1	0	0	1	1	1	1	1	8
Kanai et al. [55]	1	1	1	1	1	0	0	1	1	1	1	8
Paul et al. [60]	1	1	0	1	0	0	0	1	1	1	1	6

1 = yes; 0 = no

*tx* treatment

^a^Does not contribute points to the total score

**Table 3:** STROBE checklist for cross-sectional studies

	Item number–STROBE checklist																						
Author	1	2	3	4	5	6	7	8	9	10	11	12	13	14	15	16	17	18	19	20	21	22	Total
Prajapati et al. [56]	1	1	1	1	1	1	1	1	0	1	1	1	1	1	1	1	0	1	1	1	1	1	20/22
Taraldsen et al. [42]	1	1	1	1	1	1	1	1	0	1	1	1	1	0	1	1	0	1	1	1	1	1	19/22
Tramontano et al. [57]	1	1	1	1	1	1	1	1	0	0	1	1	1	1	1	1	1	1	1	1	1	0	19/22
Wang et al. [58]	1	1	1	1	1	0	1	1	0	0	1	1	0	1	1	1	1	1	1	1	1	1	18/22
Seo et al. [59]	1	1	1	1	0	1	1	1	0	0	1	1	0	1	1	1	1	1	0	1	1	0	16/22

1 = yes; 0 = no

**Table 4:** Study demographics

Article	Age mean ± SD (years)	Sample size	Sex (% female)	Time Post-Stroke^b^	CVA (% right hemisphere)	Assistive device use (%)
Dorsch et al. [51]	C: 65.0 ± 13.2 I: 61.8 ± 15.7	C: 73 I: 78	C: 38% I: 40%	Acute/Subacute	C: 41% I: 44%	NR
Mansfield et al. [36]	C: 61.5 ± 13^a^ I: 64 ± 19^a^	C: 28 I: 29	C: 43% I: 31%	Subacute	C: 46% (bilateral 7%) I: 38% (bilateral 7%)	Cane–C: 18%; I: 17% Rollator or wheeled walker–C: 54%; I: 52% Multiple–C: 11%; I: 3%
English et al. [52]	C: 67.8 ± 13.8 I: 65.4 ± 12.3	C: 14 I: 19	C: 36% I: 32%	Chronic	NR	Walking stick–C: 29%; I: 26% Frame–C: 7%; I: 5%
Givon et al. [53]	C: 62.0 ± 9.3 I: 56.7 ± 9.3	C: 23 I: 24	C: 29% I: 52%	Chronic	C: 67% I: 61%	NR
Danks et al. [54]	C: 58.2 ± 12.4 I: 59.1 ± 8.7	C: 14 I: 13	C: 43% I: 46%	Chronic	C: 36% I: 46%	NR
Kanai et al. [55]	C: 62.9 ± 9.1 I: 66.8 ± 10.0	C: 25 I: 23	C: 48% I: 35%	Acute/Subacute	C: 44% I: 39% (bilateral 4%)	NR
Prajapati et al. [56]	59.7 ± 15.3	16	25%	Subacute	NR	Single-point cane (50% for lab gait assessment; 25% daily use) Rollator (6% for lab assessment; 19% daily use)
Taraldsen et al. [42]	C: 46.3 ± 9.0 I: 75.2 ± 6.2	C: 10 I: 14	C: 100% I: 50%	Acute	NR	NR
Tramontano et al. [57]	68.7 ± 7.1	20	30%	Subacute	50%	None
Wang et al. [58]	63.9 ± 8.8	18	33%	Not clear (only year of diagnosis provided)	33% (bilateral 17%)	NR
Seo et al. [59]	NR	10	NR	Chronic	NR	None
Paul et al. [60]	C: 55.3 ± 12.6 I: 56.3 ± 8.7	C: 8 I: 15	C: 50% I: 53%	Chronic	C: 37% I: 53%	Walking aid–C: 38%; I: 47% Walking stick–C: 38%; I: 27% Elbow crutch(s)–I: 20%
Shin et al. [61]	55.8	6	17%	Subacute	50%	All 6 participants used assistive devices, but which type not specified

*SD* standard deviation, *CVA* cerebral vascular accident, *C* control, *I* intervention, *NR* not reported

^a^These values represent the median ± interquartile range

^b^Time post-stroke defined: Acute (1–7 days), Subacute (7 days–6 months), Chronic (> 6 months)

**Table 5:** List of included studies and data extracted from each article

Article	Wearable technology (Brand)	Location of wearable on body	Gait variables or parameters	Main Findings for Primary and Secondary Outcomes	Reliability/validity process and metrics
Dorsch et al. [51]	Triaxial accelerometers (Gulf Coast Data Concepts)	Bilateral ankles	Average daily walking time (min) Walking speed (m/s)	Feedback on 10-m walk speed plus a review of sensor-derived walking activity did not improve walking outcomes more than walking speed feedback alone [Primary: average daily walking time (p = 0.54) and 15-m walk speed (p = 0.96); Secondary: FAC scores (p = 0.39) and 3-min walking distance (p = 0.98)] Primary: No difference between groups in the rate of change in time spent walking (p = 0.32)	Process: To determine the correlation between sensor-derived walking speeds and clinical measures of walking speed Sensor-derived average daily walking speeds were highly correlated with 10-m walk speeds (r = 0.977, p < .001) Sensor-derived maximum daily speed was moderately correlated with 15-m walk speeds (r = 0.647, p < 0.001)
Mansfield et al. [36]	Triaxial accelerometers (Model X6-2mini, Gulf Coast Data Concepts)	Bilateral limbs	Walking time (min) Number of steps Average cadence (steps/min)	Primary: There was no greater increase in daily walking activity (i.e. total walking duration, number of steps) for individuals whose physiotherapists provided accelerometer-based feedback compared to those who received no feedback (p > 0.20) Secondary: Average cadence of daily walking did improve with feedback (p = 0.013)—interpreted to mean that daily walking was faster (i.e. more intense) when feedback was provided	NR
English et al. [52]	Triaxial accelerometers (activPAL3 and Actigraph GT3+)	Non-paretic hip	Stepping time (min/d) Moderate-to-vigorous physical activity (MVPA) (min/d)	Primary: This intervention was both safe and feasible Secondary: Daily siting time and prolonged sit times were reduced on average for both groups, and time spent standing and stepping increased on average; no within- or between-group effects were statistically significant Average MVPA remained very low for all participants at baseline and post-intervention	NR
Givon et al. [53]	Accelerometer (Acticial Minimitter Co.)	Hip	Steps/day	Primary: Video game intervention is feasible in a community group setting Gait speed significantly improved in each group (p = 0.04) Secondary: There was no significant change in daily steps walked as assessed by accelerometers in either group	NR
Danks et al. [54]	StepWatch Activity Monitor (Orthocare Innovations)	Non-paretic ankle	Steps/day Total walking time (h) Self-selected walking speed (m/s) Max walking speed (m/s)	Primary: A significant effect of time was observed in both groups for steps per day, total time walking, self-selected and maximal walking speed, and 6MWT distance (all p < 0.05). Subjects in the FAST + SAM group exhibited a larger increase in 6MWT distance compared to the FAST only group (p = 0.018) Results suggest that subjects with low baseline levels of walking and long-distance walking will show greater benefit when a step activity monitoring program is used in conjunction with an intervention designed to increase walking capacity	NR
Kanai et al. [55]	Fitbit One three-dimensional accelerometer (Fitbit Inc.)	Wrist	Steps/day Duration of activity (min/day)	Primary: Number of steps/day in the intervention group (i.e. use of accelerometer-based feedback plus supervised rehab) at follow-up were higher compared to the control group (supervised rehab only) (p < 0.001) Secondary: Exercise energy expenditure and duration of activity were also higher in the intervention group at follow-up compared to the control group (p ≤ 0.001) Results indicate that accelerometer-based feedback may increase physical activity, exercise energy expenditure and the duration of activity time in hospitalized patients with ischemic stroke	NR
Prajapati et al. [56]	The ABLE system (accelerometer for bilateral lower extremities): comprised 2 commercial triaxial accelerometers (Sparkfun Electronics)	Waist and bilateral ankles	Steps Cadence Number/mean of walking bouts Total walking time Total structured walking time Swing symmetry Temporal gait symmetry	Primary: On average, patients exhibited 47.5 (± 26.6) minutes of total walking time and walking duration bouts of 54.4 (± 21.5) secs during an inpatient day Secondary: A significant association was observed between the number of walking bouts and 1) total walking time (r = 0.76; p < 0.006) and 2) lab gait speed (r = 0.51; p < 0.045); and 2), as well as between slower lab gait speed and lower BBS score (r = 0.60; p < 0.013) Patients were highly variable with respect to their frequency and duration of walking activity	Process: To compare laboratory-based gait symmetry measures with wireless accelerometer-based measures of symmetry A significant difference was found between wireless accelerometer-based swing symmetry measures and lab-based measures (p = 0.006); 12 of 16 patients were more asymmetrical during the course of the day (i.e. as measured by wireless sensors
Taraldsen et al. [42]	ActivPAL single-axis accelerometer (PAL Technologies)	Sternum and bilateral thighs	Gait speed (m/s) Number of steps	Primary: Results indicate that the ActivPAL algorithms can accurately classify postures and transitions, but are not effective at detecting slow stepping. The step count algorithm is not acceptable for slow walking speeds (≤ 0.47 m/s) and needs to be improved before the ActivPAL system can be recommended for use in people who are frail Secondary: Placement of the sensor on the nonaffected leg led to less underestimation of step counts than placement on the affected leg	Process: To evaluate the concurrent validity of the ActivPAL sensor system against video observations (main objective of study)
Tramon-tano et al. [57]	Triaxial wireless accelerometer	Lumbar spine	Walking speed (m/s) Trunk acceleration	Primary: Persons with stroke demonstrated slower walking speeds than healthy adults when asked to dual task while walking (p = 0.005). There were no significant differences between groups in terms of trunk acceleration (p > 0.05); however, when controlling for walking speed, trunk acceleration was significantly different (p < 0.05), with persons with stroke exhibiting higher trunk accelerations Differences in walking speeds between the two groups was attributed to persons with stroke walking slower in hopes of trying to control abnormal trunk accelerations Secondary: A quadratic relationship between BBS score and changes in trunk acceleration RMS along the cranio-caudal axis was observed (p = 0.044)	NR
Wang et al. [58]	Textile capacitive pressure sensing insole (TCPSI) (Ajin Electronics)	Insole of shoes	Percentage of plantar pressure difference (PPD), step count, stride time, coefficient of variation, and phase coordination index (PCI)	Primary: Textile capacitive pressure sensing insoles were successfully used to analyze hemiparetic gait patterns and distinguish them from normal gait characteristics During a 40-m walk, patients with stroke had 3 × higher plantar pressure difference, lower mean plantar pressure on the affected side, a higher step count, longer stride time on the affected side, and 3 × higher PCI (indicating less balance between feet) compared to healthy controls	NR
Seo et al. [59]	Smart insole sensor	Insole of shoes	TUG, walking speed, stride length, walking time, single support time, double support time, and differences in swing and stance duration	Primary: Smart insole sensor data were similar to those calculated manually during the TUG assessment Significant differences in walking speed, stride length, TUG time, walking time, single support time, double support time, and differences in swing and stance duration were found between patients with stroke and healthy controls (p ≤ 0.005) Secondary: FMA score was significantly correlated with smart insole data (p ≤ 0.02)	NR
Paul et al. [60]	ActivPAL activity monitor (PAL Technologies) Phone accelerometer (Samsung Galaxy SIII)	ActivPAL on non-paretic leg	Steps/day Walking time (h)	The STARFISH app includes the behavior change techniques of goal setting, planning, monitoring, and feedback as well as rewards and social facilitation Primary: Using the STARFISH app for six weeks led to a significant increase in physical activity (i.e. mean number of steps/day and walking time) compared to a usual care control group (p ≤ 0.005) Secondary: Post-stroke fatigue reduced in the intervention group and increased in the control group (p = 0.003)	Process: To determine the correlation between phone accelerometer-based step counts and ActivPAL step counts A moderate correlation was found between step count data from the phone accelerometer and the ActivPAL (r = 0.67); however, at slower walking speeds the reliability of accelerometers in detecting steps is reduced
Shin et al. [61]	IMU motion sensors (XSens)	Pelvis and bilateral thighs, shanks, and feet	Amount of motion (AoM) Gait speed (m/s) Step number	Primary: Longitudinally recording joint kinematics during early gait rehabilitation post-stroke is feasible Total AoM (i.e. sum of all individual joint displacements measured), step number, number of different tasks performed during therapy, treatment intensity (i.e. change in HR), and time post-stroke were all significantly correlated with gait speed (p < 0.01, except HR p < 0.05), with total AoM revealing the greatest explained variance (R^2^ = 32.1%)	NR

*NR* not reported, *MVPA* moderate-vigorous physical activity, *6MWT* 6 min Walk Test, *FAST* fast walking training, *SAM* StepWatch activity monitor, *BBS* Berg Balance Scale, *RMS* root mean square, *TUG* Timed Up & Go, *FMA* Fugl-Meyer assessment, *IMU* inertial measurement unit, *HR* heart rate

### Quality assessment

All 13 studies were published in peer-reviewed journals; 6 were randomized control trials (RCTs) [36, 51–55], 5 were cross-sectional studies [42, 56–59], 1 was a non-randomized control trial [60], and 1 was a longitudinal pilot study [61]. In accordance with the Oxford Center for Evidence-Based Medicine (OCEBM) [62], 6 articles were ranked as Level 2 evidence, 1 as Level 3 evidence, and 6 as Level 4 evidence (Table 1). All RCTs and the non-randomized control trial were appraised using the PEDro scale, wherein all 7 were rated as high quality (PEDro score ≥ 6) (Table 2). The 5 cross-sectional studies were appraised using the STROBE checklist, which does not have a standardized scoring system; however, by examining the number of criteria each study met, a relative quality can be inferred. Of the 5 articles appraised using the STROBE checklist, they respectively met 91%, 86%, 86%, 82%, and 73% of the determined 22 criteria established to be considered the highest quality of evidence (Table 3).

### Gait/mobility analysis environment

Two studies were conducted in inpatient rehabilitation settings [36, 51], 1 study in an outpatient clinical research setting [54], and 5 studies were explicitly hospital-based [42, 55–58]. One study collected data across multiple settings including inpatient and outpatient [61]. Two studies examined data collected from participants living within their community [52, 60], and 2 studies did not state or explain the setting in which the research was conducted [53, 59] (see Table 1).

### Participant characteristics

The mean sample size for included studies was 23 (range 6–78 participants). All studies except one [59] reported the age of participants: persons with stroke had a mean age ≥ 55 years of age, and one study reported a mean age below that of 46.3 years for a healthy control group [42]. Amongst the studies that reported the gender of participants [36, 42, 51–58, 60, 61], the ratio of men to women varied greatly. Regarding chronicity of stroke, 1 study included participants with acute stroke (0–7 days) [42], 4 included sub-acute (7 days–6 months) [36, 56, 57, 61], 2 included both acute and subacute [51, 55], and 5 included chronic (> 6 months) [52–54, 59, 60]. Nine studies reported where the stroke occurred and/or the side of the body that was affected and included participants with both left and right-sided strokes or bilateral stroke [36, 51, 53–55, 57, 58, 60, 61]. Of the 5 studies that reported on the use of assistive devices by participants [36, 52, 56, 60, 61], a variety of assistive devices were used when walking including single point canes, rollators, and walking sticks (see Table 4 for more details).

### Wearable technologies used

Studies employed an array of wearable technologies in order to assess gait and mobility post-stroke. The most commonly used devices were accelerometers [36, 42, 51–57, 60, 61]. One study reported the use of a smartphone application for real-time assessment of step count [60]. Two studies conducted assessments with foot pressure sensors [58, 59].

### Parameters of gait and mobility assessed

Measures of gait and mobility included spatiotemporal parameters as well as measures of physical activity (Table 5). The most widely assessed spatiotemporal parameters of gait were gait speed [42, 51, 54, 57, 59, 61] and cadence [36, 56]. Less common parameters included single limb support time, double limb support time, stride time, stride length, swing symmetry and temporal gait symmetry [56, 58, 59]. The two most commonly reported measures of mobility were step count [36, 42, 53–56, 58, 60, 61] and duration of physical activity (e.g. time spent walking/active) [36, 51, 52, 54–56, 60]. Less commonly reported measures included levels of moderate-to-vigorous physical activity [52] and number of mean walking bouts [56]. One study examined foot plantar pressure distribution during walking [58] while another study evaluated lower body kinematic changes during walking early post-stroke [59]. None of the studies analyzed associations between patient-reported outcomes (e.g., QOL, measures of fatigue) and wearable technology-based measures of gait and mobility.

## Discussion

The interest in the use of wearable technology such as sensors has sky rocketed in recent years. Researchers and healthcare providers have begun to recognize the potential depth, breadth, and ease of data collection that the emergence of such technology can enable. In examining the use of wearable technology to assess gait and mobility post-stroke, the majority of the studies captured in this systematic review were randomized control trials of high quality [36, 51–55] and cross-sectional studies [42, 56–59]. Studies varied in age of participants [36, 42, 51–58, 60, 61], time post-stroke [36, 42, 51–57, 59–61], location of stroke [36, 51, 53–55, 57, 58, 60, 61], gender of participants [36, 42, 51–58, 60, 61], and the use of assistive devices [36, 52, 56, 57, 59–61]. Five research studies assessed their participants’ gait and mobility pre/post intervention or hospital stay, with data collection times of 2–3 consecutive days [53, 55] up to 1 week [52, 54, 60]. Two research studies conducted their data collection across the length of participants’ inpatient rehabilitation stay (which varied from a few days to approximately 3 weeks) [36, 51], and one study recorded data across 12 treatment sessions during inpatient/outpatient rehabilitation [61]. Three studies used wearable technology to examine parameters of gait during clinical walking assessments [57–59], and two studies collected data on one day for either ≤ 1 h [42] or 8 consecutive hours [56]. As assistive devices (such as canes) can compensate for lower limb weakness, impaired balance and force production often caused by hemiplegia and/or paresthesia post-stroke, approximately half of the studies documented assistive device use and specifics during walking assessment trials [36, 52, 56, 57, 59–61]. Due to the limited number of studies conducted on wearable technology assessment of gait and mobility post-stroke, such variation in participant characteristics was expected.

### Environmental settings typically used in gait and mobility research post-stroke

Gait and mobility are important parameters that inform a patient’s ability to remain independent and engage in the community. Our systematic literature review shows that while wearable technology has been extensively used in gait and mobility research post-stroke, assessment of community mobility using wearable devices is limited. Although wearable technology was primarily developed to eliminate barriers of laboratory-based research, our research reveals that this technology has been underutilized especially in diagnosing gait and mobility restrictions after stroke in the community and more natural environments. Surprisingly, the community-based studies that used wearable technology to address mobility after stroke focused on interventions [52, 60]. Thus, the applicability of such research seems questionable as evidence related to diagnostic utilization of wearable devices in this patient population has not yet been firmly established. Our literature review recommends future original research should first focus on systematic evaluation of key diagnostic metrics of gait and mobility by using wearable technology in a community-based setting. This approach warrants the need to determine the type of wearable technology which is appropriate for a community-based evaluation of gait and mobility in persons with stroke. Once the appropriate wearable device(s) has been established, the next logical step is to utilize them as a diagnostic tool, assessing community mobility deficits post-stroke and ultimately devising novel treatment options. However, the current lack of diagnostic utilization of wearables for chronic stroke requires further study including knowledge and skills around interpretation of wearable data and translation of wearable data to actionable measures for improving gait and mobility among rehabilitation providers.

### Type of wearable device(s) used in gait and mobility research post-stroke

As anticipated, there is little consistency in the choice of device used to collect and analyze people’s gait and mobility post-stroke. The most commonly used wearable technologies were triaxial accelerometers of varied brands [36, 51, 52, 55–57, 61], with fewer studies using pressure sensors for gait assessment [58, 59]. While devices such as the StepWatch activity monitor have proven to be valid and reliable in post-stroke populations [6, 63], demonstrating their utility in research, the cost of such devices and associated software may be prohibitive for widespread use in clinical practice. Other accelerometer-based wearable technologies, such as smartphones and Fitbits, which are more commercially available, less expensive, and user friendly, may be more practical for patient and clinician use. Many of these devices collect and record a multitude of mobility parameters (i.e. single limb stance time, acceleration, physical activity level, etc.) versus just a single measure. Most devices used are manufactured so that they are small, lightweight and wireless, allowing users to wear them in a variety of settings while not being intrusive. Devices in this systematic review were most commonly worn at the hip [52, 53, 56], thigh [42, 61], and ankle [51, 54, 56] to allow capturing of gait and mobility parameters of interest. Overall, this review stresses the need to devise wearable technology that is affordable, light weight, user-friendly and at the same time accurately captures complex mobility deficits that a person with stroke might encounter in day-to-day life and the community.

### Outcomes investigated in gait and mobility research post-stroke

Our findings suggest that gait speed [42, 53, 54, 57, 59, 61] and cadence [36, 56] are the two most widely assessed spatiotemporal parameters of gait via wearable technology. Considering that gait speed is the sixth vital sign and a major predictor of quality of life and functional status within a community [24], it is not surprising that gait speed was the most commonly assessed gait parameter in post-stroke populations. It is also recognized, however, that gait speed may not reflect the full functional picture of ambulators post-stroke. Therefore, assessing additional measures of mobility is pertinent for elucidating the impact of stroke on walking function. The most common measures of mobility included step count [36, 42, 53–56, 58, 60, 61] and duration of activity [36, 51, 52, 54–56, 60], which were collected and examined across all settings. Step count was assessed using a variety of accelerometers, including smartphone applications; duration of physical activity was primarily assessed via accelerometers. Due to current limitations in wearable technology and relevant research, our knowledge on various important parameters such as quality and efficiency of gait, functionality of gait, and impact of gait and mobility deficits on quality of life and long-term health outcomes in people with stroke is limited.

### Reliability and validity of wearable devices in gait and mobility research post-stroke

While the efficacy of technology utilization in post-stroke populations is important to highlight and understand, only a limited number of studies in this systematic review examined and reported on reliability and validity metrics. Thus, the consistency and accuracy of various measures and outcome tools used in majority of the research studies that were included in this review were unknown. The studies that reported on validity and reliability used both uni- (e.g., ActivPAL) and tri-axial accelerometers [42, 51, 56, 60]. These studies conducted reliability and validity analysis of one or more of the following outcome measures: gait speed, step counts, and/or swing symmetry, which were compared against a criterion standard that included one of the following: 3D gait analysis, clinical outcome measures of gait and mobility, or video-based counts. Thus, our review emphasizes the need for future research to specifically examine validity and reliability metrics of wearable devices used to measure gait and mobility deficits in persons post-stroke. Particularly, abnormal movement and force production patterns that are commonly seen in this population more so amplifies the need to utilize a wearable technology that accurately assesses these parameters while maintaining reliability and validity.

### Clinical applicability of wearable technology to improve walking post-stroke

This systematic review highlights the potential of wearable technologies for use in clinical practice. Clinical and home-based assessments provide a simplistic snapshot of a patient’s functional mobility, whereas wearable technologies can provide real-time vital data to provide insights as to their patients’ lived experiences (e.g., time spent active versus sedentary, time spent walking, number of steps taken, time spent in specific activities). These data can help clinicians design interventions more tailored to an individual’s needs by capturing barriers to mobility that cannot be otherwise assessed in the clinic, as well as guide preventative measures and best practices. Additionally, many of the commonly used devices also allow for the collection of location-based data that may permit clinicians to examine measures of participation and life-space as well, expanding opportunities for clinicians to directly address outcomes that are meaningful to persons with chronic stroke.

## Limitations

There are a number of limitations that should be considered when interpreting the results of our study. First, the results of the methodological quality assessments included in the systematic review are based on the assessors interpretation of the quality of the articles. Second, our search did not include keywords pertaining to “reliability” or “validity” of wearable technology as this was not considered in our initial search strategy. Therefore, had such terms been included there may have been more findings related to these metrics. Our results are also limited to the available MeSH headings and chosen keywords for this study. Moreover, given the relatively small number of studies conducted on wearable technologies and gait assessment post-stroke and the large number of research questions pertaining to the subject, it is difficult to make strong recommendations about the type of wearable technologies best suited to assess gait and mobility in post-stroke populations. Lastly, due to the shortcoming of validity of consumer-grade devices for assessing gait and mobility in neurologically involved populations, there is a lack of accurate algorithms to monitor and account for variability in gait and mobility patterns. Future research is needed to examine the validity of different consumer wearables during free-living walking and mobility assessments in persons with stroke.

## Conclusion

Wearable technologies have the capacity to provide information on gait analysis in real-world settings, which allows the ability to assess and address mobility limitations such as reduced walking speed/endurance and reduced physical activity within different environments (e.g., home/community, indoor/outdoor). The current systematic review found that relevant research over the past decade has primarily been conducted in lab-based or hospital settings. Gait speed is the most commonly captured spatiotemporal parameter of gait and step count is the most commonly captured mobility metric, assessed primarily via triaxial accelerometers. Future research should be conducted within more community settings, as well as examine associations between patient-reported outcomes and wearable technology-based measures of gait and mobility (e.g., walking speed, time spent walking, intensity of activity) to provide a richer understanding of the impact of stroke and rehabilitation on patients’ lives. Lastly, our results showed a limited number of studies that examined reliability and validity of wearable devices, highlighting the need for more studies to examine psychometric properties of these devices when collecting gait and mobility information in persons post-stroke. These studies are essential to determine which wearable technologies are most effective to utilize and in which contexts they are most appropriate.

## Supplementary Information

**Additional file 1.** Appendix.

## Data Availability

Data sharing is not applicable to this article as no datasets were generated or analyzed during the current study.

## Abbreviations

QOL: Quality of life
PRISMA: Preferred Reporting Items for Systematic Reviews and Meta-Analyses
PEDro: Physiotherapy Evidence Database
STROBE: Strengthening the Reporting of Observational Studies in Epidemiology
RCTs: Randomized control trials
OCEBM: Oxford Center for Evidence-Based Medicine
