# Supplementary material for: Utilization of wearable technology to assess gait and mobility post-stroke: a systematic review
Source: J Neuroeng Rehabil. 2021 Apr 21;18:67. doi: 10.1186/s12984-021-00863-x (PMC8059183; doi:10.1186/s12984-021-00863-x)
Supplement: Supplementary file 1 — Additional file 1. Appendix. [file 12984_2021_863_MOESM1_ESM.docx]

**Appendix**

We used the following search terms with exact search strings for Ovid MEDLINE, CINAHL and Cochrane library databases:

**Ovid MEDLINE**

*Total number of articles retrieved = 67*

| **#** | **Query** | **Results** |
| --- | --- | --- |
| 1 | exp Stroke/ | *141,975* |
| 2 | Stroke Rehabilitation/ | *14,144* |
| 3 | stroke*.kf,tw. | *267,145* |
| 4 | ("cerebrovascular accident*" or CVA).kf,tw. | *9,267* |
| 5 | Cerebrovascular Disorders/ | *46,853* |
| 6 | 1 or 2 or 3 or 4 or 5 | *337,377* |
| 7 | Wearable Electronic Devices/ | *3,655* |
| 8 | Fitness Trackers/ | *709* |
| 9 | exp Cell Phone/ | *16,704* |
| 10 | Monitoring, Ambulatory/ | *8,334* |
| 11 | exp Accelerometry/ | *9,718* |
| 12 | ("wear* activit* tracker*" or "wear* electronic device*" or "wear* diagnos* device*" or "wear* comput* device*").kf,tw. | *523* |
| 13 | ("fitness tracker*" or "activit* tracker*").kf,tw. | *826* |
| 14 | ("cell* telephone*" or "cell* phone*" or "mobile phone*" or "mobile telephone*" or "text* messag*").kf,tw. | *16,887* |
| 15 | (acceleromet* or "ambulatory monitor*" or "outpatient monitor*").kf,tw. | *20,904* |
| 16 | exp Computers, Handheld/ | *9,127* |
| 17 | (microcomput* or smartphone*).kf,tw. | *22,764* |
| 18 | ("inertial measure* unit*" or IMU or gyroscop* or "smart watch*" or pedomet* or GPS or "smart phone*" or smartphone*).kf,tw. | *48,158* |
| 19 | 7 or 8 or 9 or 10 or 11 or 12 or 13 or 14 or 15 or 16 or 17 or 18 | *109,383* |
| 20 | "Activities of Daily Living"/ | *66,206* |
| 21 | ("activit* of daily living" or adl*).kf,tw. | *37,534* |
| 22 | exp Exercise/ | *205,897* |
| 23 | (exercis* or "physical activit*" or walk* or resist* or aerobic* or endur* or ambulat*).kf,tw. | *1,745,008* |
| 24 | exp Gait/ | *30,461* |
| 25 | gait*.kf,tw. | *54,975* |
| 26 | 20 or 21 or 22 or 23 or 24 or 25 | *1,896,248* |
| 27 | 6 and 19 | *1,796* |
| 28 | 26 and 27 | *788* |
| 29 | limit 28 to (meta analysis or randomized controlled trial or "systematic review") | *93* |
| 30 | limit 29 to english language | *91* |
| 31 | Robotics/ | *21,521* |
| 32 | Exoskeleton Device/ | *798* |
| 33 | (robotic* or exoskeleton*).kf,tw. | *40,126* |
| 34 | 31 or 32 or 33 | *48,825* |
| 35 | 30 not 34 | *88* |
| 36 | limit 35 to yr="2010 - 2020" | *67* |

**CINAHL**

*Total number of articles retrieved = 37*

| **#** | **Query** | **Limiters/Expanders** | **Results** |
| --- | --- | --- | --- |
| S1 | "stroke" OR "stroke rehabilitation" OR "cerebrovascular disorders" OR "cerebrovascular accident" OR "cva"  (cerebrovascular accident) | Search modes -  Boolean/Phrase | *127,043* |
| S2 | "wearable electronic devices" OR "fitness trackers" OR "cell phone" OR "wear activity trackers" OR "wear electronic device" OR "wear diagnostic device" OR "wear computer device" OR "activity tracker" OR "cell telephone" OR "mobile phone" OR "mobile telephone" OR "monitoring/ambulatory" OR "accelerometry" OR "computers, handheld" OR "accelerometer" OR "ambulatory monitor" OR "outpatient monitor" OR "microcomputer" OR "smartphone" OR "inertial measure unit" OR “imu” (inertial measurement unit) OR “gyroscope” OR “smart watch” OR “pedometer” OR “gps” (global positioning system) | Search modes -  Boolean/Phrase | *23,626* |
| S3 | “gait” OR “activities of daily living” OR “exercise” OR “adl” (activities of daily living) OR “physical activity” OR “walk”, OR “resistance” OR “aerobic” OR “endurance” OR “ambulation” | Search modes -  Boolean/Phrase | *415,874* |
| S4 | “robotics” OR  “exoskeleton” | Search modes -  Boolean/Phrase | *8,995* |
| S5 | S1 AND S2 AND S3 | Search modes -  Boolean/Phrase | *268* |
| S6 | S5 NOT S4 | Limiters - Published Date: 20100101-20200931; English Language; Publication Type: Meta Analysis, Randomized Controlled Trial, Systematic Review  Search modes - Boolean/Phrase | *37* |

**Cochrane Library**

*Total number of articles retrieved = 250*

| **ID** | **Search Hits** | **Results** |
| --- | --- | --- |
| #1 | MeSH descriptor: [Stroke] explode all trees | *10,194* |
| #2 | Stroke Rehabilitation | *12,782* |
| #3 | (stroke*):ti,ab,kw | *57,704* |
| #4 | ("cerebrovascular accident*" or CVA):ti,ab,kw | *13,253* |
| #5 | MeSH descriptor: [Cerebrovascular Disorders] explode all trees | *15,672* |
| #6 | #1 or #2 or #3 or #4 or #5 | *66,146* |
| #7 | MeSH descriptor: [Wearable Electronic Devices] this term only | *87* |
| #8 | MeSH descriptor: [Fitness Trackers] this term only | *104* |
| #9 | MeSH descriptor: [Cell Phone] explode all trees | *1,786* |
| #10 | MeSH descriptor: [Monitoring, Ambulatory] this term only | *554* |
| #11 | MeSH descriptor: [Accelerometry] explode all trees | *965* |
| #12 | ("wear* activit* tracker*" or "wear* electronic device*" or "wear* diagnos* device*" or "wear* comput* device*"):ti,ab,kw | *1* |
| #13 | ("cell* telephone*" or "cell* phone*" or "mobile phone*" or "mobile telephone*" or "text* messag*"):ti,ab,kw | *3,398* |
| #14 | ("cell phone*" or "cell telephone*" or "mobile phone*"):ti,ab,kw | *3,341* |
| #15 | (acceleromet* or "ambulatory monitor*" or "outpatient monitor*"):ti,ab,kw | *4,261* |
| #16 | (microcomput* or smartphone*):ti,ab,kw | *4,914* |
| #17 | ("inertial measure* unit*" or IMU or gyroscop* or "smart watch*" or pedomet* or GPS or "smart phone*" or smartphone*):ti,ab,kw | *9,188* |
| #18 | #7 or #8 or #9 or #10 or #11 or #12 or #13 or #14 or #15 or #16 or #17 | *17,911* |
| #19 | #6 and #18 | *621* |
| #20 | MeSH descriptor: [Activities of Daily Living] explode all trees | *9,518* |
| #21 | (adl* or "activit* of daily living"):ti,ab,kw | *4,184* |
| #22 | MeSH descriptor: [Exercise] explode all trees | *25,218* |
| #23 | (exercis* or "physical activit*" or walk* or resist* or aerobic* or endur* or ambulat*):ti,ab,kw | *211,265* |
| #24 | MeSH descriptor: [Gait] explode all trees | *2,075* |
| #25 | (gait*):ti,ab,kw | *10,262* |
| #26 | #20 or #21 or #22 or #23 or #24 or #25 | *223,743* |
| #27 | #19 and #26 | *302* |
| #28 | #27 with Publication Year from Jan 2010 to Sep 2020 | *250* |
